# Diterpenoids and triterpenoids from the roots of *Tripterygium regelii* and their inhibitory effects on colorectal cancer cells

**DOI:** 10.3389/fchem.2026.1784072

**Published:** 2026-02-20

**Authors:** Ping Guo, Jianming Yu, Qi Zhao, Jintao Sun, Rongxin Lin, Linlin Sui, Aijing Leng, Chao Wang, Shuo Wang, Dawei Li

**Affiliations:** 1 Dalian Medical University, Dalian, China; 2 Dalian Fusheng Natural Medicine Development Co., Ltd, Dalian, China; 3 The First Affiliated Hospital of Dalian Medical University, Dalian, China

**Keywords:** colorectal cancer cells, diterpenoids, ferroptosis, *Tripterygium regelii*, triterpenoids

## Abstract

A total of 22 terpenoids including one new triterpenoid (**1**), two new diterpenoids (**2** and **3**), and 19 known compounds (**4**–**22**) were isolated from the roots of *Tripterygium regelii*. Their structures were identified using NMR and HRESIMS techniques. Antiproliferative activity screening assays revealed that the new compound **2**, along with several known compounds, exhibits potential anti-colorectal cancer efficacy. Further investigations employing flow cytometry, immunofluorescence staining and electron microscopy demonstrated that compound **2** induces the generation of reactive oxygen species (ROS). This, in turn, promotes lipid peroxidation and mitochondrial damage, ultimately enhancing ferroptosis in colorectal cancer cells. These findings underscore the value of compound **2** as a potential candidate for anti-tumor drug development.

## 1 Introduction

**FIGURE 1:**
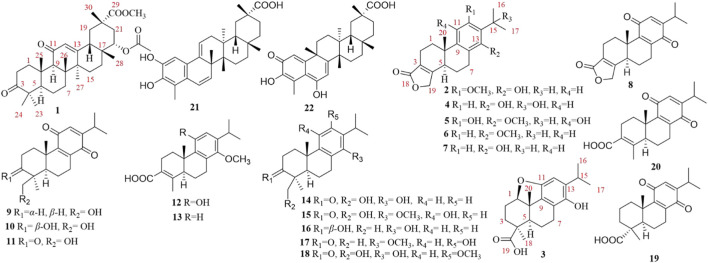
Chemical structures of compounds **1**–**22**.

The genus *Tripterygium* (Celastraceae), endemic to East Asia, comprises three species: *Tripterygium wilfordii*, *Tripterygium hypoglaucum*, and *Tripterygium regelii* (*T. regelii*) (Lv et al., 2019). These plants have been used for centuries in traditional Chinese medicine to treat autoimmune and inflammatory diseases, including rheumatoid arthritis, nephrotic syndrome, and systemic lupus erythematosus (Lü et al., 2015; Lv et al., 2019; Zhang et al., 2021). Over 500 secondary metabolites, primarily sesquiterpenoids, diterpenoids, triterpenoids, flavonoids, and lignans, have been identified from this genus (Lv et al., 2019). Among the three species, *T. regelii is* distributed mainly in northeastern China, Japan, and Korea (Lv et al., 2019) and remains relatively understudied compared to its well-investigated congeners. In our previous work, we isolated ten dihydro-*β*-agarofuran sesquiterpenoids and twenty-three macrolide sesquiterpene pyridine alkaloids from the roots of *T. regelii*, both of which demonstrated anti-inflammatory activities (Li et al., 2025; Sun et al., 2023). Notably, previous studies have reported that diterpenoids and triterpenoids from *T. regelii* exhibit anticancer activities (Fan et al., 2016; Fan et al., 2017; Jiang et al., 2018). Especially, celastrol, a key active triterpenoid found in *Tripterygium* species including *T. regelii*, has demonstrated potent and broad-spectrum cytotoxicity against numerous malignancies (Pilipović et al., 2023; Wang et al., 2023). As part of our ongoing research into the bioactive constituents of *T. regelii*, we focused on the isolation and structural characterization of its diterpenoids and triterpenoids, and the evaluation of their cytotoxicity. This effort led to the isolation of 22 terpenoids (Figure 1), including one new triterpenoid (**1**) and two new diterpenoids (**2** and **3**). The results of preliminary antiproliferative activity screening using the colorectal cancer cell line HT29 revealed that several known diterpenoids and triterpenoids (**6**, **8**–**10**, **16**, **17**, **21**, and **22**) exhibited significant cytotoxicity (inhibition rate >70% at 50 μM). More importantly, the new diterpenoid **2** also demonstrated potent antiproliferative activity. However, we prioritized **2** for further mechanistic investigation not only for its potent activity, but also because its mechanism of action remained completely unexplored as a newly identified diterpenoid. Subsequent mechanistic studies using flow cytometry, electron microscopy, and pathway protein analysis confirmed that compound **2** exerts anti-colorectal cancer effects by inducing ferroptosis. These findings provide a deeper mechanistic insight into the inhibitory activity against colorectal cancer of *T. regelii* and enrich the pharmacological basis for its application.

## 2 Experiment

### 2.1 Apparatus

NMR spectra (Bruker Avance 600 MHz, Bruker BioSpin AG, Fällanden, Switzerland), HRESIMS data (AB SCIEX X500R QTOF, AB SCIEX LLC, Framingham, MA, United States), and analytical HPLC (Thermo Scientific Dionex UltiMate 3000, Thermo Fisher Scientific Inc., Waltham, MA, United States) were obtained using the respective instruments. Preparative HPLC was performed on a SEP LC-52 system from Separation (Beijing) Technology Co., Ltd. (Beijing, China), equipped with a MWD UV detector. A YMC Pack ODS-A C18 column (250 × 10 mm, 5 μm, YMC Co., Ltd., Kyoto, Japan) was used for separation. Column chromatography was performed using aluminum oxide (Shanghai Macklin Biochemical Co., Ltd., Shanghai, China), silica gel (200–300 mesh; Qingdao Marine Chemical Inc., Qingdao, China), and ODS (Tianjin Bonna-Agela Technologies Co., Ltd., Tianjin, China). Chromatographic-grade methanol (Sigma-Aldrich Co., LLC, St. Louis, MO, United States) and other analytical-grade solvents (Tianjin Kermel Chemical Reagent Co., Ltd., Tianjin, China) were used.

### 2.2 Materials

The roots of *T. regelii* were collected on 7 July 2019 from Ji’an City, Jilin Province, China. The roots were identified by Professor Chao Wang and deposited at Dalian Medical University.

### 2.3 Extraction and isolation

*T. regelii* roots (30 kg) were extracted with 70% ethanol using heat reflux extraction (3 × 2 h). Following ethanol evaporation, the residual aqueous solution was diluted with water and subjected to sequential liquid-liquid partitioning using petroleum ether, EtOAc, and *n*-BuOH. The resulting EtOAc extract was divided into two portions: 20 g and 400 g. The petroleum ether extract was fractionated into 18 fractions (SG1–SG18) via silica gel column chromatography, eluting with gradient EtOAc/CH_3_OH mixtures (20:1, 10:1, 5:1, 2:1, 1:1, 1:100). Fraction SG14 was further purified by preparative HPLC at 3 mL/min using a 58:42 CH_3_CN/H_2_O mixture (0.03% TFA) as the eluent, yielding compound **21** (6.8 mg). The 20 g portion of the EtOAc extract was subjected to silica gel column chromatography and eluted with a gradient of CH_2_Cl_2_/CH_3_OH (100:0, 50:1, 20:1, 10:1, 1:1, 1:5, 1:10, 0:100), resulting in the isolation of 8 fractions designated D1 to D8. Fractions D2 and D3 were combined and further separated via ODS medium-pressure column chromatography using a CH_3_OH/H_2_O gradient elution system, which afforded 14 subfractions labeled DR1 to DR14. Subfractions DR6 to DR12 were merged and further purified by preparative HPLC at a flow rate of 3 mL/min. The eluent used was a 50:50 mixture of CH_3_OH and H_2_O containing 0.03% trifluoroacetic acid (TFA). This purification step yielded eleven compounds, namely compounds **3** (4.5 mg), **7** (10.5 mg), **10** (5.0 mg), **12** (7.0 mg), **13** (4.2 mg), **14** (3.3 mg), **15** (4.2 mg), **18** (6.6 mg), **20** (12.5 mg), **19** (8.7 mg), and **22** (5.0 mg). The 400 g portion of the EtOAc extract was fractionated into 4 fractions by aluminum oxide column chromatography, using a series of EtOAc/CH_3_OH eluent mixtures with gradient ratios of 100:1, 4:1, 1:1, and 1:100. Fraction 2 was further sub-fractionated into 26 fractions (designated E1–E26) via silica gel column chromatography, eluting with CH_2_Cl_2_/CH_3_OH mixtures in gradient proportions of 100:1, 50:1, 15:1, 10:1, and 5:1. Subsequently, sub-fractions E5–E6 were subjected to additional silica gel column chromatography, employing petroleum ether/EtOAc mixtures (100:1, 20:1, 10:1, 8:1, 5:1, 2:1, 1:100) as the eluent, which resulted in the isolation of 24 sub-fractions (ED1–ED24). Sub-fractions ED16–ED24 were purified by preparative HPLC (equipped with an RP C18 column) at a flow rate of 3 mL/min. The eluent used was a 70:30 MeOH/H_2_O mixture containing 0.03% TFA (v/v), yielding compounds **1** (1.4 mg), **5** (2.3 mg), and **6** (4.5 mg). Sub-fractions ED11–ED15 were similarly purified by preparative HPLC (RP C18 column, 3 mL/min) using an 80:20 MeOH/H_2_O mixture (0.03% TFA, v/v) as the eluent, affording compounds **4** (5.6 mg), **16** (3.8 mg), and **17** (2.2 mg). Fractions E7–E16 were combined and further purified by preparative HPLC at a flow rate of 3 mL/min. The elution was performed with a 60:40 MeOH/H_2_O mixture containing 0.03% TFA, yielding four compounds: **2** (18.3 mg), **8** (4.8 mg), **9** (13.6 mg), and **11** (5.5 mg).

#### 2.3.1 Methyl 3,11-dioxo-22*α*-acetoxy-olean-12-en-29-oate (1)

White solid; ^1^H NMR (CDCl_3_, 600 MHz) and ^13^C NMR (CDCl_3_, 150 MHz) data see Table 1; HRESIMS *m/z* 541.3486 [M + H]^+^ (calcd. for C_33_H_49_O_6_^+^, 541.3524).

**TABLE 1:** The ^1^H NMR (600 MHz) and ^13^C NMR (150 MHz) data of compounds **1**–**3** (*δ* in ppm, *J* in Hz).

No.	1^a^		2^b^		3^b^	
	*δ*_H_	*δ*_C_	*δ*_H_	*δ*_C_	*δ*_H_	*δ*_C_
1	2.94 m 1.38 m	39.9	2.55 dd (13.2,6.0) 1.69 m	32.6	3.70 dd (12.6, 3.6)	99.6
2	2.63 m 2.37 m	34.3	2.43 m 2.35 m	19.1	2.31 m 1.96 m	25.9
3	​	217.2	​	125.2	2.56 m 1.27 m	38.3
4	​	47.9	​	166.7	​	46.2
5	1.30 m	55.5	2.71 overlap	42.7	1.44 dd (12.6, 3.6)	51.1
6	1.56 m	18.9	2.02 m 1.89 m	20.7	2.23 m 2.17 m	22.6
7	1.73 m 1.45 overlap	32.2	2.81 dd (16.8, 7.2) 2.73 overlap	23.8	2.93 m 2.50 m	24.9
8	​	45.5	​	116.6	​	120.4
9	2.44 s	61.3	​	144.8	​	137.9
10	​	36.8	​	37.6	​	42.0
11	​	199.3	6.54 s	101.3	​	153.3
12	5.66 s	129.1	​	158.3	6.49 s	106.9
13	​	167.2	​	122.5	​	134.9
14	​	43.8	​	154.1	​	146.7
15	1.78 m 1.26 m	25.9	3.50 m	25.8	3.31 m	28.2
16	1.91 m 1.54 m	20.1	1.26 d (7.2)	21.3	1.14 d (6.6)	23.9
17	​	37.1	1.27 d (7.2)	21.2	1.14 d (6.6)	23.5
18	2.28 dd (13.8, 3.0)	46.6	​	176.9	1.26 s	27.6
19	2.21 t (13.8) 1.45 overlap	38.5	4.93 d (16.2) 4.86 d (16.2)	72.4	​	181.1
20	​	42.9	1.04 s	22.5	0.92 s	14.6
21	1.87 m 1.80 m	33.4	​	​	​	​
22	4.87 dd (12.0, 4.8)	76.5	​	​	​	​
23	1.11 s	26.6	​	​	​	​
24	1.07 s	21.6	​	​	​	​
25	1.26 s	15.8	​	​	​	​
26	1.17 s	18.6	​	​	​	​
27	1.42 s	23.8	​	​	​	​
28	0.93 s	24.8	​	​	​	​
29	​	177.4	​	​	​	​
30	1.33 s	20.5	​	​	​	​
22-OAc	2.05 s	170.6 21.3	​	​	​	​
29-OCH_3_	3.68 s	52.3	​	​	​	​
12-OCH_3_	​	​	3.76 s	55.9	​	​

^a^ Measured in CDCl_3_.

^b^ Measured in CD_3_OD.

#### 2.3.2 12-Methoxytriptophenolide (2)

White solid; ^1^H NMR (CD_3_OD, 600 MHz) and ^13^C NMR (CD_3_OD, 150 MHz) data see Table 1; HRESIMS *m/z* 343.1898 [M + H]^+^ (calcd. for C_21_H_27_O_4_^+^, 343.1904).

#### 2.3.3 1,11-Epoxy-13-isopropyl-14-hydroxy-podocarpa-8,11,13-trien-19-oic acid (3)

White solid; ^1^H NMR (CD_3_OD, 600 MHz) and ^13^C NMR (CD_3_OD, 150 MHz) data see Table 1; HRESIMS *m/z* 331.1887 [M + H]^+^ (calcd. for C_20_H_27_O_4_^+^, 331.1904).

### 2.4 Cell line and cell culture

The colorectal cancer cell lines HCT116 and HT29 used in this study were obtained from the American Type Culture Collection (ATCC) and maintained in stable passage in our laboratory. Cells were cultured in Dulbecco’s Modified Eagle Medium (DMEM; Gibco) supplemented with 10% fetal bovine serum (FBS; Gibco) and 5% antibiotic-antimycotic solution (Solarbio). Cultures were maintained in a humidified incubator at 37 °C with 5% CO_2_, and cell passaging was performed every 3 days.

### 2.5 Cell viability assay

The cytotoxic effect of compounds isolated from *T. regelii* were assessed using a cell viability assay, with detailed procedures as previously described (Chen, et al., 2025). Briefly, colorectal cancer cells in the logarithmic growth phase were seeded into 96-well plates at 3,000 cells per well. Following cell adhesion, the cells were treated with drug-supplemented medium or solvent control for 48 h, and cell proliferation in each group was measured via the CCK-8 assay.

### 2.6 Colony formation

HCT116 or HT29 were trypsinized to form single-cell suspensions and seeded into 6-well plates at 500–1,000 per well. The cells were then treated with medium supplemented with various concentrations of compound **2** or solvent control for 10–14 days. Following treatment, cells were fixed with a fixative solution (methanol:glacial acetic acid:water = 1:1:8) for 10 min, then stained with 1% crystal violet for 10 min. Excess crystal violet was washed away with PBS to visualize colony formation across all groups.

### 2.7 Lipid peroxidation assay

Cells were seeded in 6-well plates and treated with compound **2** for 48 h, after which the subsequent assay was conducted. First, the BODIPY 581/591 C11 probe (Biyuntian, Cat. No. S0043M) was diluted at a ratio of 1:1000 in DMEM medium to prepare the probe working solution. Next, the cells were trypsinized, centrifuged, and rinsed once with PBS; the PBS was then discarded. For each tube of cell pellets, 200 µL of the diluted probe solution was added, followed by incubation at 37 °C for 30 min. Post-incubation, the samples were centrifuged to remove the supernatant, and the cell pellets were washed twice with PBS. Finally, the cells were resuspended in 500 µL of PBS and analyzed via flow cytometry using a BD C6 Plus instrument, with the experimental results processed and analyzed using Flow Jo software.

### 2.8 Mitochondrial damage detection

Cells were seeded in 6-well plates and treated with compound **2** for 48 h prior to the subsequent assay. First, cells were harvested via centrifugation; after removing the culture medium, they were resuspended and thoroughly mixed in electron microscopy (EM) fixative, followed by fixation at 4 °C for 2–4 h. The supernatant was then discarded, and 0.1 M phosphate buffer (PB, pH 7.4) was added. The mixture was gently agitated for a 3-min rinse, then centrifuged, and this washing procedure was repeated three times. Next, the samples were embedded in a 1% agarose solution. After fixation with 1% osmium tetroxide at room temperature for 2 h (protected from light), the samples were rinsed three times with 0.1 M PB (pH 7.4), with each rinse step lasting 15 min. Dehydration was performed by sequentially immersing the samples in a graded ethanol series (30% → 50% → 70% → 80% → 95% → 100% → 100%), with each immersion lasting 20 min. This was followed by two 15-min soaks in 100% acetone. For infiltration, the samples were incubated in an embedding agent mixture (acetone:812 resin = 1:1) at 37 °C overnight, then soaked in pure 812 resin at 37 °C for 5–8 h. The samples were then placed into embedding molds, incubated in an oven at 37 °C overnight, and polymerized at 60 °C for 48 h. Ultrathin sections (60–80 nm) were cut, then stained with a saturated 2% uranyl acetate alcoholic solution for 8 min (protected from light). After staining, the sections were rinsed three times with 70% ethanol and three times with ultrapure water in sequence. Subsequently, they were stained with a 2.6% lead citrate solution for 8 min (shielded from carbon dioxide), rinsed three more times with ultrapure water, and dried at room temperature overnight. Finally, the sections were observed under a transmission electron microscope (JEM1400PLUS), and images were captured for further analysis.

### 2.9 ROS detection

Cells in the growth phase, which had been cultured on coverslips, were seeded into 24-well plates. Following treatment with compound **2** for 24 h, the subsequent assay was performed. Briefly, the original culture medium was aspirated, and the cells were rinsed twice with PBS. The DCFH-DA fluorescent probe (Biyuntian, Cat. No. S0033M) was diluted in PBS at a ratio of 1:1000, and 500 µL of this diluted probe solution was added to each well. After incubating at 37 °C for 30 min, the solution in each well was removed, and any residual diluted probe was washed away with PBS. An anti-fluorescence quenching agent was then added for coverslip mounting, and images were acquired using an inverted fluorescence microscope (Olympus, Model 1X73) to obtain the assay results.

### 2.10 Western blot

HCT116 and HT29 cell pellets treated with compound **2** were collected by centrifugation. Total proteins were extracted using RIPA lysis buffer, with concentrations quantified via BCA assay. Following separation by SDS-PAGE, proteins were transferred to PVDF membranes. Membranes were probed with primary antibodies against SLC7A11 (AWA00502, Abiowell), SLC3A2 (AWA61227, Abiowell), GPX4 (AWA11352, Abiowell), and GAPDH (81640-5-RR, Proteintech), where GAPDH served as a loading control to ensure equal protein loading. Proteins were visualized by autoradiography and quantified using ImageJ software.

## 3 Results and discussion

### 3.1 Isolated phytochemicals from *Tripterygium regelii*

**FIGURE 2:**
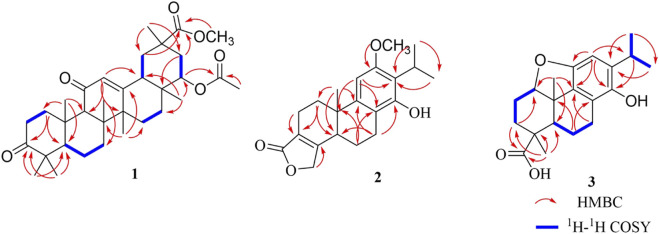
Main HMBC and ^1^H– ^1^H COSY correlations of compounds **1–3.**

**FIGURE 3:**
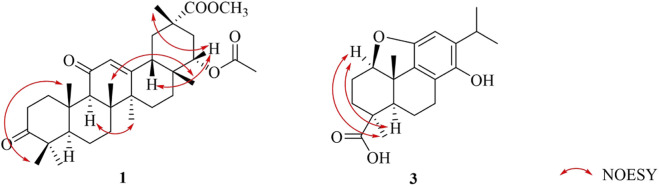
Key NOESY correlations of compounds **1** and **3**.

Compound **1** was obtained as a white solid. Its molecular formula was established as C_33_H_48_O_6_, corresponding to 10 degrees of unsaturation, based on HRESIMS data showing a peak at *m/z* 541.3486 [M + H]^+^ (calcd. for C_33_H_49_O_6_^+^, 541.3524) and supported by NMR analysis (Table 1). The ^1^H NMR spectrum of **1** indicated the presence of eight methyl groups [*δ*_H_ 1.11 (s, H_3_-23), 1.07 (s, H_3_-24), 1.26 (s, H_3_-25), 1.17 (s, H_3_-26), 1.42 (s, H_3_-27), 0.93 (s, H_3_-28), 1.33 (s, H_3_-30), 2.05 (s, OCOCH_3_)], a methoxyl group [*δ*_H_ 3.68 (s, COOCH_3_)], one oxymethine group [*δ*_H_ 4.87 (dd, *J* = 12.0, 4.8 Hz, H-22)], a trisubstituted double bond [*δ*_H_ 5.66 (s, H-12)] (Table 1). The ^13^C NMR spectrum displayed 33 carbon resonances, comprising two ketone carbonyls [*δ*_C_ 217.2 (C-3) and 199.3 (C-11)], two ester carbonyls [*δ*_C_ 177.4 (C-29) and 170.6 (22-OCOCH_3_)], two olefinic carbons [*δ*_C_ 129.1 (C-12) and 167.2 (C-13)], and one oxygenated carbon [*δ*_C_ 76.5 (C-22)] (Table 1). Analysis of the 1D NMR data suggested that **1** is an oleanane-type triterpenoid with a planar structure closely resembling that of 22*β*-acetylglabric acid (Shen et al., 1995). However, key differences were observed: the hydroxyl group at C-3 and the COOH group at C-29 in 22*β*-acetylglabric acid were replaced by a carbonyl group and a COOCH_3_ group, respectively, in **1** (Figure 2). These modifications were corroborated by 2D NMR experiments (Figure 2). In the HMBC spectrum, correlations between OCH_3_-29 and C-29, together with the chemical shift of C-29 (*δ*_C_ 177.4), confirmed the presence of COOCH_3_ group (Figure 2). Additionally, the ketone at C-3 was assigned based on HMBC correlations from H_3_-23 and H_3_-24 to C-3, along with its characteristic chemical shift (*δ*_C_ 217.2, C-3) (Figure 2). The ^1^H–^1^H COSY spectrum revealed several contiguous proton spin systems: H_2_-1/H_2_-2, H-5/H_2_-6/H_2_-7, H_2_-15/H_2_-16, H-18/H_2_-19, and H_2_-21/H-22 (Figure 2). The relative configuration of **1** was elucidated via NOESY experiments, which showed key correlations between H_3_-24 and H_3_-25, H-9 and H_3_-27, H_3_-26 and H_3_-28, H-18 and H-22, and H_3_-30 and H-22 (Figure 3). Accordingly, the structure of **1** was determined as methyl 3,11-dioxo-22*α*-acetoxy-olean-12-en-29-oate, as depicted in Figure 1.

Compound **2** was obtained as a white solid. Its molecular formula was determined to be C_21_H_26_O_4_ based on HRESIMS analysis, which showed an ion peak at*m/z* 343.1898 [M + H]^+^ (calcd. for C_21_H_27_O_4_^+^, 343.1904). The ^1^H NMR spectrum of **2** (in CD_3_OD) displayed signals consistent with a pentasubstituted aromatic ring [*δ*_H_ 6.54 (s, H-11)], an oxymethylene group [*δ*_H_ 4.93 (d, *J* = 16.2 Hz, H-19a) and 4.86 (d, *J* = 16.2 Hz, H-19b)], a methoxyl group [*δ*_H_ 3.76 (s, OCH_3_-12)], an isopropyl unit [*δ*_H_ 3.50 (m, H-15), 1.26 (d, *J* = 7.2 Hz, H_3_-16), and 1.27 (d, *J* = 7.2 Hz, H_3_-17)], and an angular methyl group [*δ*_H_ 1.04 (s, H_3_-20)] (Table 1). The ^13^C NMR and HSQC spectra revealed 21 carbon resonances (Table 1), classified into four methyls, five methylenes, three methines, and nine quaternary carbons. The NMR data of **2** closely resembled those of the known compound triptophenolide (Zeng et al., 2013), with a key difference: the hydrogen atom at C-12 in triptophenolide was replaced by a methoxyl group in **2**. This substitution was confirmed by the HMBC correlation from OCH_3_-12 to C-12, supported by the chemical shift of C-12 (*δ*_C_ 158.3) (Figure 2). Therefore, the structure of **2** was established as 12-methoxytriptophenolide.

Compound **3** was assigned the molecular formula C_20_H_26_O_4_ based on HRESIMS data, which exhibited an ion peak at *m/z* 331.1887 [M + H]^+^ (calcd. for C_20_H_27_O_4_^+^, 331.1904). The ^1^H NMR spectrum of **3** (in CD_3_OD) displayed signals corresponding to a pentasubstituted aromatic ring [*δ*_H_ 6.49 (s, H-12)], an isopropyl unit [*δ*_H_ 3.31 (m, H-15),1.14 (d, *J* = 6.6 Hz, H_3_-16), and 1.14 (d, *J* = 6.6 Hz, H_3_-17)], an oxymethine group [*δ*_H_ 3.70 (dd, *J* = 12.6, 3.6 Hz, H-1)], and two angular methyl groups [*δ*_H_ 1.26 (s, H_3_-18) and 0.92 (s, H_3_-20)] (Table 1). The ^13^C NMR spectrum revealed 20 carbon signals, including an oxygenated carbon (*δ*_C_ 99.6), a carboxyl carbon (*δ*_C_ 181.1), and an aromatic group (*δ*_C_ 106.9, 120.4, 134.9, 137.9, 146.7, 153.3) (Table 1). These spectroscopic features indicated that **3** is structurally related to (1*R*,5*S*,10*S*)-1,11-oxyferruginol (Zhao et al., 2015). Further comparison of the NMR data between **3** and (1*R*,5*S*,10*S*)-1,11-oxyferruginol revealed several key differences: the hydroxyl group at C-12 in the reference compound was replaced by a hydrogen atom in **3**, the hydrogen at C-14 was replaced by a hydroxyl group, and the methyl group at C-19 was oxidized to a carboxyl group (*δ*_C_ 181.1, C-19) (Figure 2). In the HMBC spectrum, correlations from H-12 to C-9, C-11, and C-14, along with the chemical shifts of C-12 (*δ*_C_ 106.9) and C-14 (*δ*_C_ 146.7), supported the presence of a hydroxyl group at C-14 (Figure 2). Additionally, the carboxyl group at C-19 was confirmed by HMBC correlations from H-3 and H_3_-18 to C-19 (Figure 2). The relative configurations of the carboxyl group and H-1 were elucidated as *β* and *α*-oriented, respectively, based on the correlations of H-1/H-5 and H-1/H_3_-18 in the NOESY spectrum (Figure 3). Thus, the structure of compound **3** was determined to be 1,11-epoxy-13-isopropyl-14-hydroxy-podocarpa-8,11,13-trien-19-oic acid.

Additionally, nineteen known compounds (**4**−**22**) were isolated from *T. regelii*, and their structures were elucidated by comparison of NMR spectroscopic data with those reported in the literature: triptobenzene E (**4**) (Takaishi et al., 1997), isoneotriptophenolide (**5**) (Zeng et al., 2013), triptophenolide methyl ether (**6**) (Deng et al., 1982), triptophenolide (**7**) (Zeng et al., 2013), quinone-21 (**8**) (Morota et al., 1995a), triptoquinone D (**9**) (Shishido et al., 1994), triptoquinone C (**10**) (Shishido et al., 1994), triptoquinone B (**11**) (Shishido et al., 1994), triptobenzene H (**12**) (Li et al., 1997), triptinin-A (**13**) (Xu et al., 1997), triptobenzene A (**14**) (Jiang et al., 2019), triptonoterpenol (**15**) (Zeng et al., 2013), triptobenzene B (**16**) (Takaishi et al., 1997), wilforol E (**17**) (Morota et al., 1995a), wilforol F (**18**) (Morota et al., 1995a), 8,12-dienabieta-11,14-dione-19-acid (**19**) (Ma et al., 2010), triptoquinone A (**20**) (Takaishi et al., 1992), wilforol B (**21**) (Morota et al., 1995b), and 6-hydroxy-celastrol (**22**) (Zhang et al., 2014).

### 3.2 Antiproliferative activity against colorectal cancer cells of isolated compounds

**FIGURE 4:**
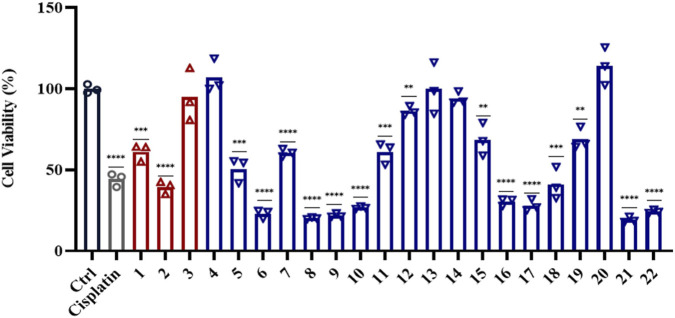
The cytotoxicities of compounds **1**-**22** against HT29 cell line following 24-h exposure at 50 μM.

**FIGURE 5:**
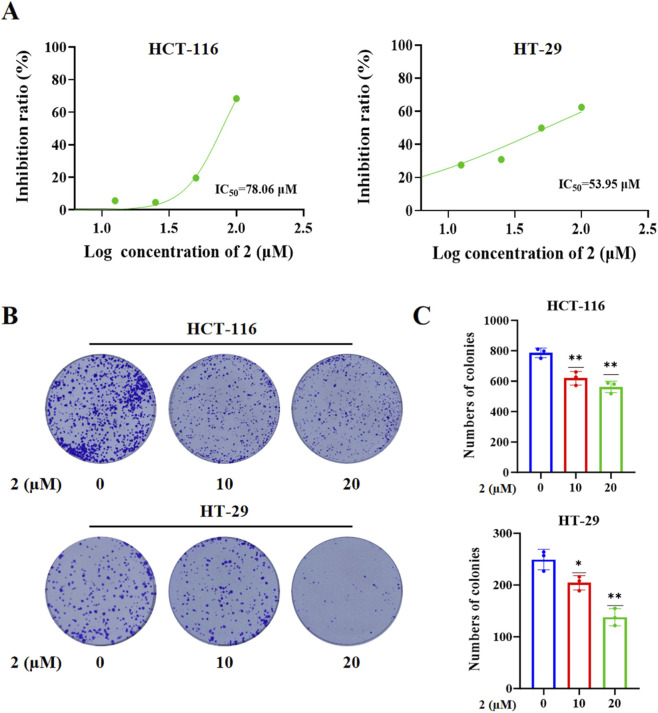
Compound **2** inhibited the proliferation of colorectal cancer cells. **(A)** The concentration-dependent inhibitory effect of compound **2** on HCT116 and HT29 cells; **(B)** The reduction in cloning formation induced by compound **2**; **(C)** The numbers of colonies per well were calculated. **2** (µM): the concentration of compound **2** (µM).

Previous studies have indicated that diterpenoids and triterpenoids from *T. regelii* possess antitumor potential (Fan et al., 2016; Fan et al., 2017; Jiang et al., 2018; Wang et al., 2023). Employing the human colorectal cancer cell line HT29 as the *in vitro* experimental model, we assessed the antitumor potential of the 22 isolated terpenoid compounds through cell viability assays, with particular emphasis on investigating the antiproliferative activity of the newly identified compounds. Our screening results indicated that among the three newly identified compounds (**1**–**3**), compound **2** exhibited moderate antiproliferative activity against colorectal cancer *in vitro*, whereas compounds **1** and **3** showed weak or no detectable activity under the same experimental conditions (Figure 4). Overall, compounds **22**, **6**, **8**–**10**, **16**, **17**, **21**, and **22** exhibited antiproliferative effects against colorectal cancer cells (Figure 4). To further characterize the antiproliferative potency of compound **2**, we determined its IC_50_ values in two colorectal cancer cell lines (HCT116 and HT29), as shown in Figure 5A. After 48 h of treatment, compound **2** exhibited moderate inhibitory effects on CRC cells HCT116 and HT29 proliferation, with IC_50_ values of 53.95 μM and 78.06 μM in the two cell lines, respectively. Additionally, colony formation assays further confirmed that compound **2** displays acceptable anti-colorectal cancer activity under prolonged exposure, as illustrated in Figures 5B,C.

**FIGURE 6:**
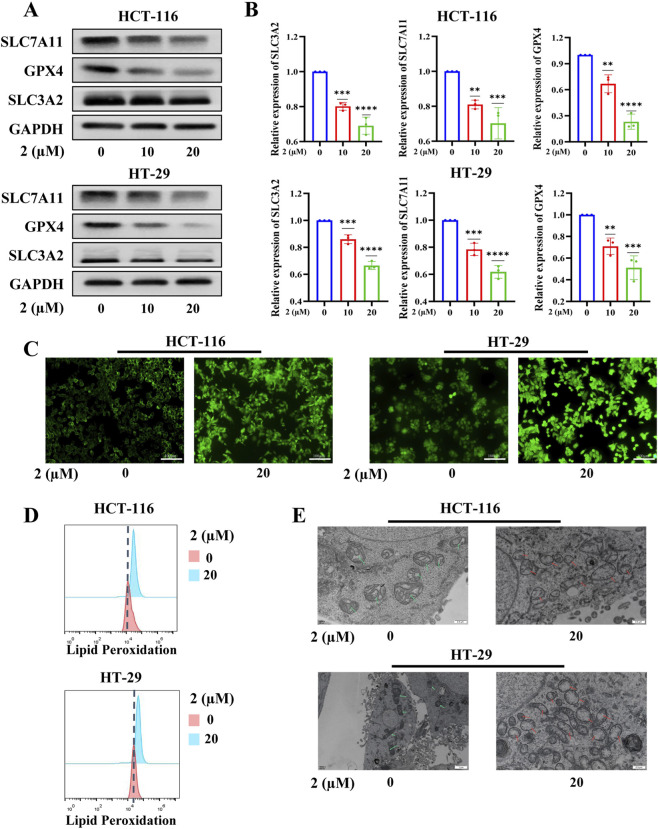
Compound **2** inhibited the proliferation of colorectal cancer cells by inducing ferroptosis. **(A)** Western blotting demonstrated that compound **2** promotes the expression of ferroptosis-related proteins in HCT116 and HT29; **(B)** The quantification of **(A)**; **(C)** The reactive oxygen species (ROS) levels in each group were detected using the DCFH-DA fluorescent probe; **(D)** Lipid peroxidation levels in each group were detected by flow cytometry following staining with the BODIPY 581/591 C11 probe; **(E)** Transmission electron microscopy revealed the degree of mitochondrial damage in each group. **2** (µM):the concentration of compound **2** (µM).

We were also interested in elucidating the mechanism by which compound **2** induces colorectal cancer cell death. Given that inducing ferroptosis is an important anti-tumor strategy, we first examined whether compound **2** inhibits tumor cell proliferation via the ferroptosis pathway (Chen et al., 2021; Zhou et al., 2024). Western blot analysis initially confirmed that compound **2** exerts anti-colorectal cancer effects by inducing ferroptosis (Figures 6A,B). To investigate the role of ROS-mediated lipid peroxidation and mitochondrial damage, key drivers of ferroptosis (Cao et al., 2023; Niu et al., 2021), we utilized an ROS probe (DCFH-DA for general ROS) and fluorescence imaging. This approach revealed that compound 2 promotes ROS production in colorectal cancer cells. (Figure 6C). Furthermore, flow cytometry with a lipid peroxidation probe confirmed that compound **2** induces lipid peroxidation (Figure 6D). Additionally, transmission electron microscopy demonstrated that compound **2** induces mitochondrial damage in colorectal cancer cells (Figure 6E). Taken together, these findings indicate that compound **2** exerts anti-colorectal cancer activity by inducing ferroptosis.

## 4 Conclusion

A total of 22 terpenoids including one new triterpenoid (**1**), two new diterpenoids (**2** and **3**), and nineteen known compounds (**4**–**22**) were isolated from the *T. regelii* roots. Their structures were identified using NMR and HRESIMS techniques. What’s more, compound **2**, one of the new isolates, was screened and validated to inhibit colorectal cancer cell growth by inducing ferroptosis. This process is driven by the induction of reactive oxygen species (ROS), which in turn promotes lipid peroxidation and mitochondrial damage. Overall, these findings not only enrich the chemical constituents of *T. regelii*, but also identify compound **2** as an encouraging compound for the development of anti-colorectal cancer drugs targeting the ferroptosis pathway. This study underscores the significant value of natural products in modern drug discovery.

## Data Availability

The original contributions presented in the study are included in the article/Supplementary Material, further inquiries can be directed to the corresponding authors.
