# Supplementary material for: Diterpenoids and triterpenoids from the roots of Tripterygium regelii and their inhibitory effects on colorectal cancer cells
Source: Front Chem. 2026 Feb 20;14:1784072. doi: 10.3389/fchem.2026.1784072 (PMC12962659; doi:10.3389/fchem.2026.1784072)
Supplement: Supplementary file 1 [file DataSheet1.docx]

**Diterpenoids and triterpenoids from the roots of *Tripterygium regelii* and their inhibitory effects on colorectal cancer cells**

Ping Guo^1†^, Jianming Yu^1†^, Qi Zhao^1^, Jintao Sun^1^, Rongxin Lin^2^, Linlin Sui^1^, Aijing Leng^3^, Chao Wang^1,3^, Shuo Wang^2*^ and Dawei Li^1,2,3*^

^1^ Dalian Medical University, Dalian, China

^2^ Dalian Fusheng Natural Medicine Development Co ., Ltd, Dalian, China

^3^ The First Affiliated Hospital of Dalian Medical University, Dalian, China

*CORRESPONDENCE

E-mail addresses:

Shuo Wang, [wangshuo_1983@qq.com](mailto:wangshuo_1983@qq.com)

Dawei Li, [lidw87@163.com](mailto:lidw87@163.com)

^†^These authors contributed equally to this work and share first authorship

**Table of Contents**

**[Figure S1](#_Toc10207)**[.](#_Toc10207) ^[1](#_Toc10207)^[H NMR (600 MHz, CDCl](#_Toc10207)_[3](#_Toc10207)_[) spectrum of the compound](#_Toc10207) **[1](#_Toc10207)**[. 3](#_Toc10207)

**[Figure S2](#_Toc29511)**[.](#_Toc29511) ^[13](#_Toc29511)^[C NMR (150 MHz, CDCl](#_Toc29511)_[3](#_Toc29511)_[) spectrum of the compound](#_Toc29511) **[1](#_Toc29511)**[. 3](#_Toc29511)

**[Figure S3](#_Toc471)**[. HSQC spectrum of the compound](#_Toc471) **[1](#_Toc471)**[. 4](#_Toc471)

**[Figure S4](#_Toc14526)**[. HMBC spectrum of the compound](#_Toc14526) **[1](#_Toc14526)**[. 4](#_Toc14526)

**[Figure S5](#_Toc3445)**[.](#_Toc3445) ^[1](#_Toc3445)^[H-](#_Toc3445)^[1](#_Toc3445)^[H COSY spectrum of the compound](#_Toc3445) **[1](#_Toc3445)**[. 5](#_Toc3445)

**[Figure S6](#_Toc32636)**[.NOESY spectrum of the compound](#_Toc32636) **[1](#_Toc32636)**[. 5](#_Toc32636)

**[Figure S7](#_Toc916)**[. HRESIMS spectrum of the compound](#_Toc916) **[1](#_Toc916)**[. 6](#_Toc916)

**[Figure S8](#_Toc10416)**[.](#_Toc10416) ^[1](#_Toc10416)^[H NMR (600 MHz, CD](#_Toc10416)_[3](#_Toc10416)_[OD) spectrum of the compound](#_Toc10416) **[2](#_Toc10416)**[. 6](#_Toc10416)

**[Figure S9](#_Toc9573)**[.](#_Toc9573) ^[13](#_Toc9573)^[C NMR (150 MHz, CD](#_Toc9573)_[3](#_Toc9573)_[OD) spectrum of the compound](#_Toc9573) **[2](#_Toc9573)**[. 7](#_Toc9573)

**[Figure S10](#_Toc10905)**[. HSQC spectrum of the compound](#_Toc10905) **[2](#_Toc10905)**[. 7](#_Toc10905)

**[Figure S11](#_Toc10927)**[. HMBC spectrum of the compound](#_Toc10927) **[2.](#_Toc10927)** [8](#_Toc10927)

**[Figure S12](#_Toc13895)**[. HRESIMS spectrum of the compound](#_Toc13895) **[2](#_Toc13895)**[. 8](#_Toc13895)

**[Figure S13](#_Toc32547)**[.](#_Toc32547) ^[1](#_Toc32547)^[H NMR (600 MHz, CD](#_Toc32547)_[3](#_Toc32547)_[OD) spectrum of the compound](#_Toc32547) **[3](#_Toc32547)**[. 9](#_Toc32547)

**[Figure S14](#_Toc29722)**[.](#_Toc29722) ^[13](#_Toc29722)^[C NMR (150 MHz, CD](#_Toc29722)_[3](#_Toc29722)_[OD) spectrum of the compound](#_Toc29722) **[3](#_Toc29722)**[. 9](#_Toc29722)

**[Figure S15](#_Toc12100)**[. HSQC spectrum of the compound](#_Toc12100) **[3](#_Toc12100)**[. 10](#_Toc12100)

**[Figure S16](#_Toc21891)**[. HMBC spectrum of the compound](#_Toc21891) **[3](#_Toc21891)**[. 10](#_Toc21891)

**[Figure S17](#_Toc10686)**[.](#_Toc10686) ^[1](#_Toc10686)^[H-](#_Toc10686)^[1](#_Toc10686)^[H COSY spectrum of the compound](#_Toc10686) **[3](#_Toc10686)**[. 11](#_Toc10686)

**[Figure S18](#_Toc11674)**[.NOESY spectrum of the compound](#_Toc11674) **[3](#_Toc11674)**[. 11](#_Toc11674)

**[Figure S19](#_Toc24592)**[. HRESIMS spectrum of the compound](#_Toc24592) **[3](#_Toc24592)**[. 12](#_Toc24592)


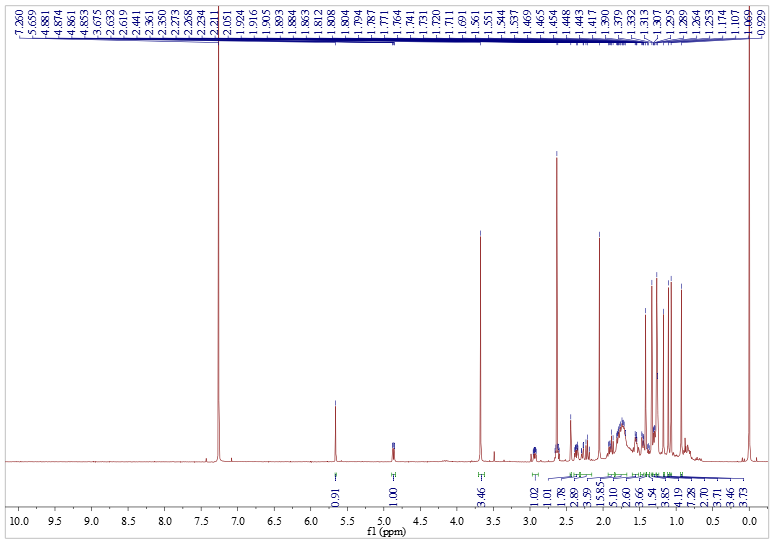


**Figure S1.** ^1^H NMR (600 MHz, CDCl_3_) spectrum of the compound **1.**


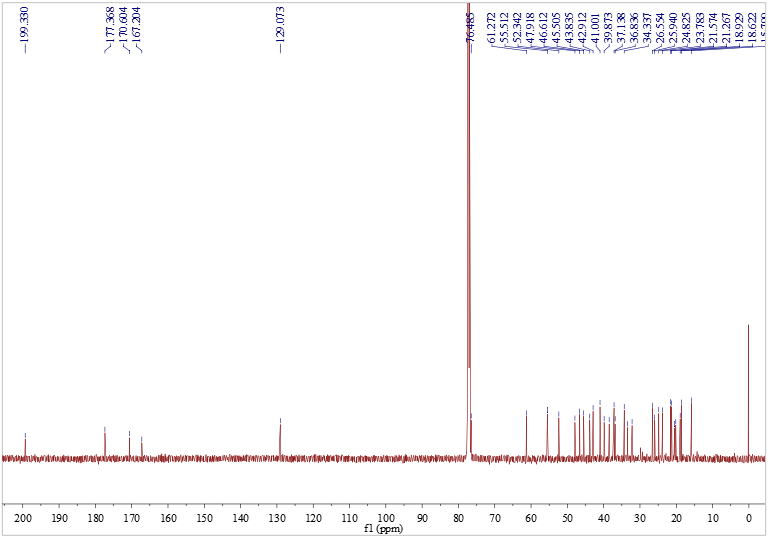


**Figure S2.** ^13^C NMR (150 MHz, CDCl_3_) spectrum of the compound **1.**


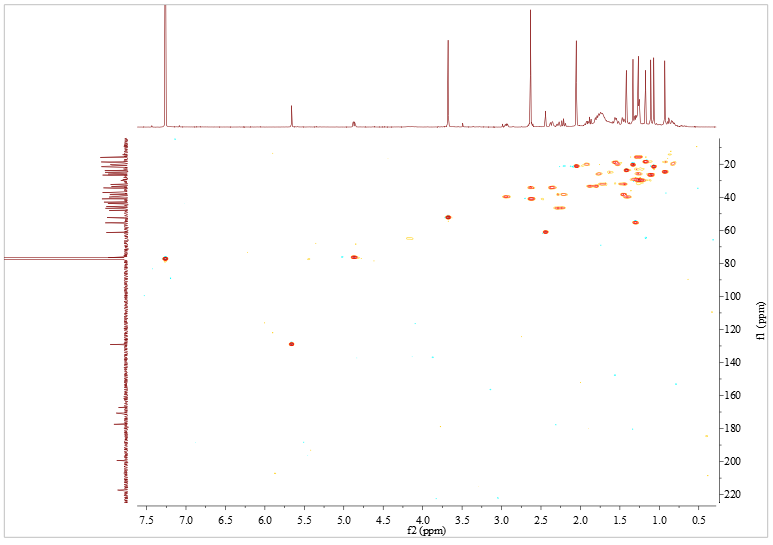


**Figure S3.** HSQC spectrum of the compound **1.**


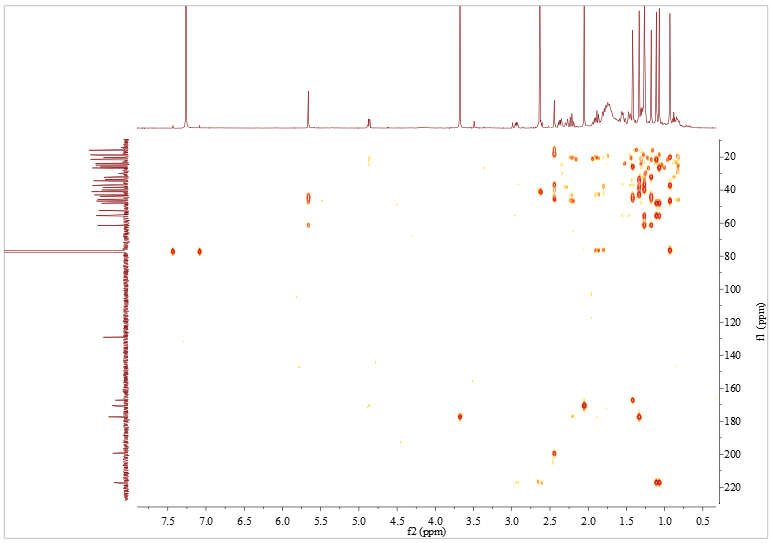


**Figure S4.** HMBC spectrum of the compound **1.**


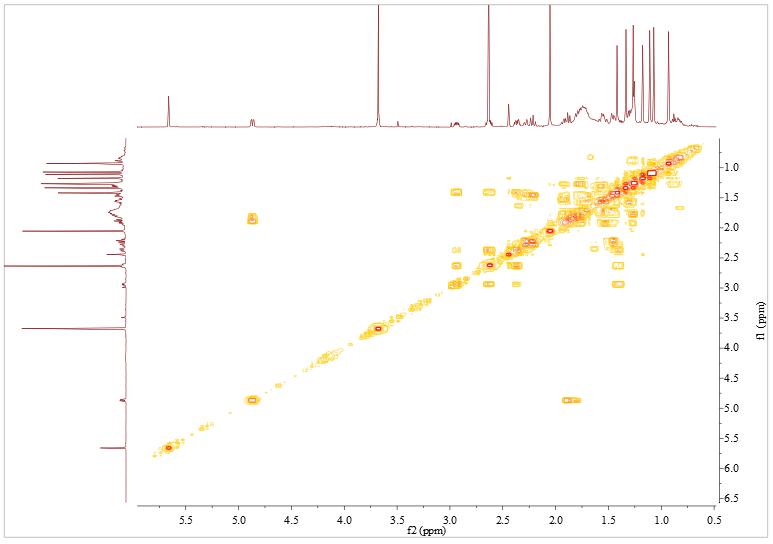


**Figure S5.** ^1^H-^1^H COSY spectrum of the compound **1.**


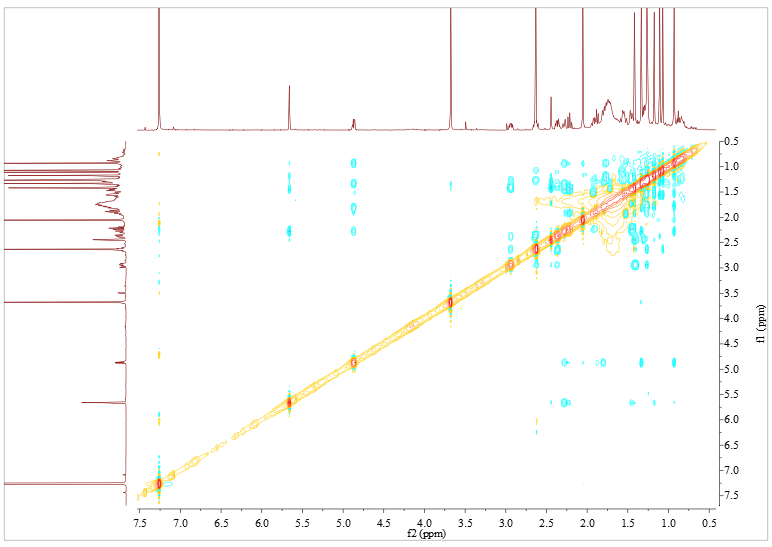


**Figure S6.**NOESY spectrum of the compound **1.**

**Figure S7.** HRESIMS spectrum of the compound **1.**


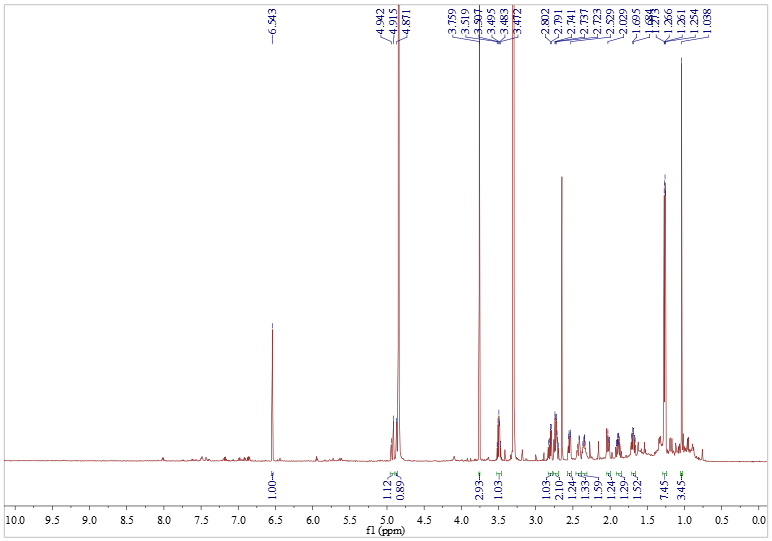


**Figure S8.** ^1^H NMR (600 MHz, CD_3_OD) spectrum of the compound **2.**


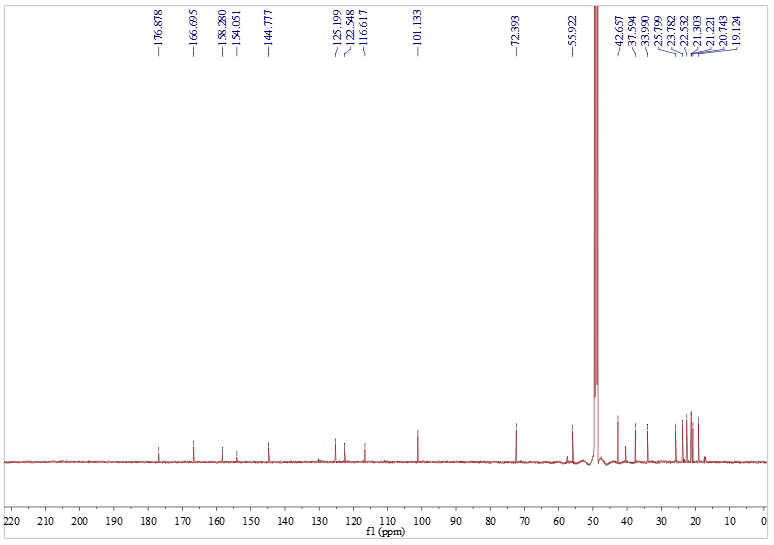


**Figure S9.** ^13^C NMR (150 MHz, CD_3_OD) spectrum of the compound **2.**


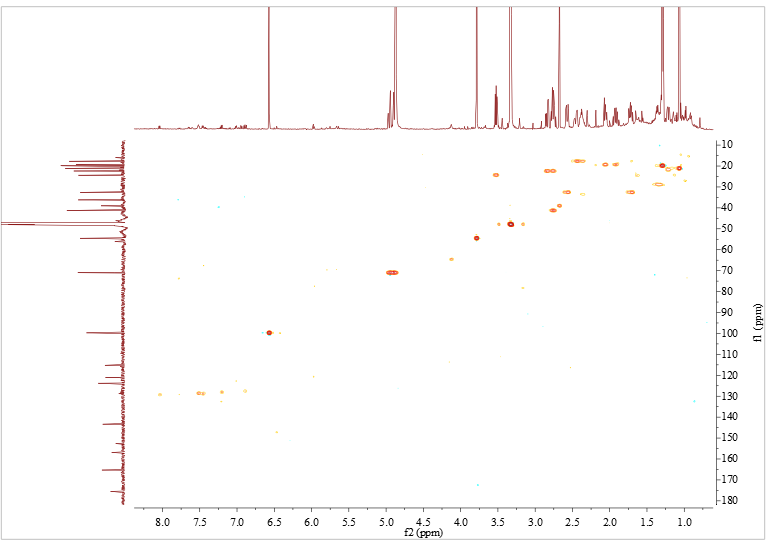


**Figure S10.** HSQC spectrum of the compound **2.**


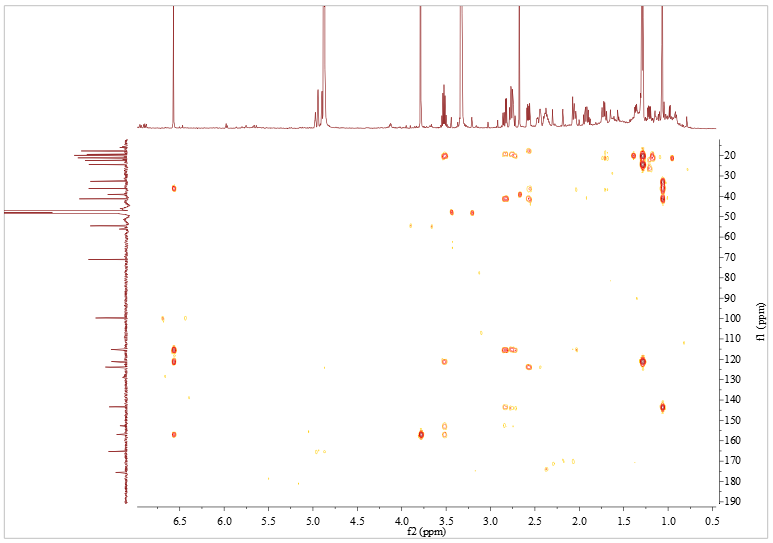


**Figure S11.** HMBC spectrum of the compound **2.**

**Figure S12.** HRESIMS spectrum of the compound **2.**


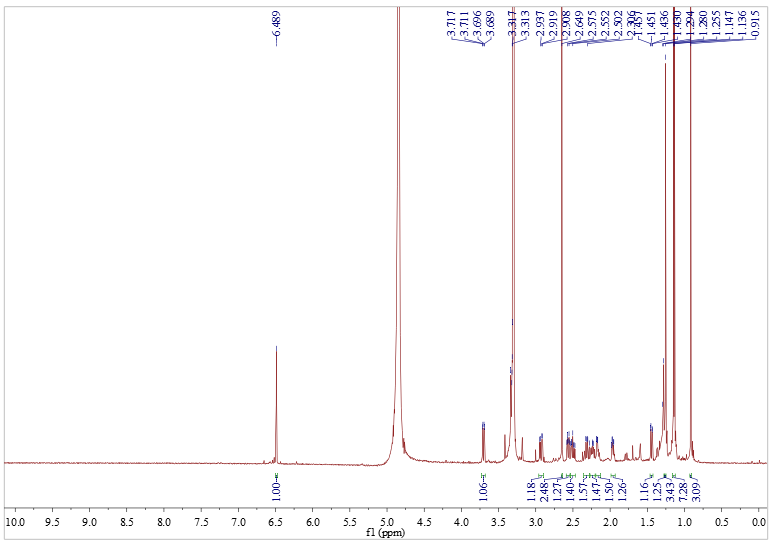


**Figure S13.** ^1^H NMR (600 MHz, CD_3_OD) spectrum of the compound **3.**


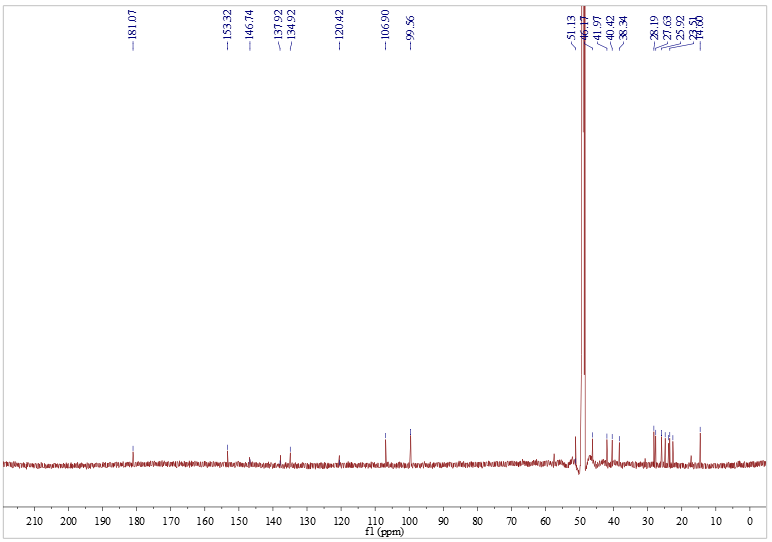


**Figure S14.** ^13^C NMR (150 MHz, CD_3_OD) spectrum of the compound **3.**


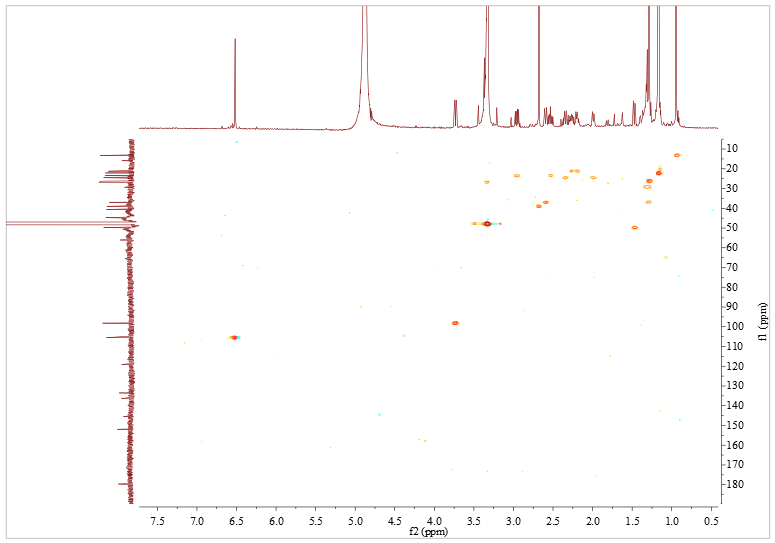


**Figure S15.** HSQC spectrum of the compound **3.**


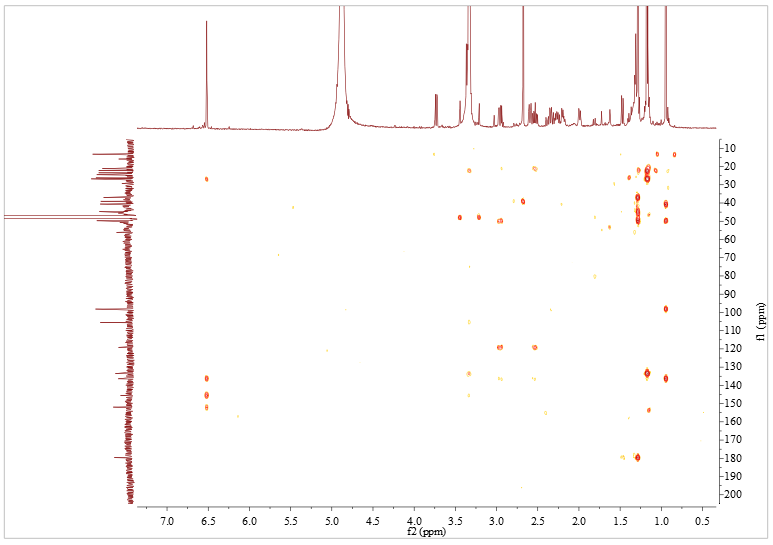


**Figure S16.** HMBC spectrum of the compound **3.**


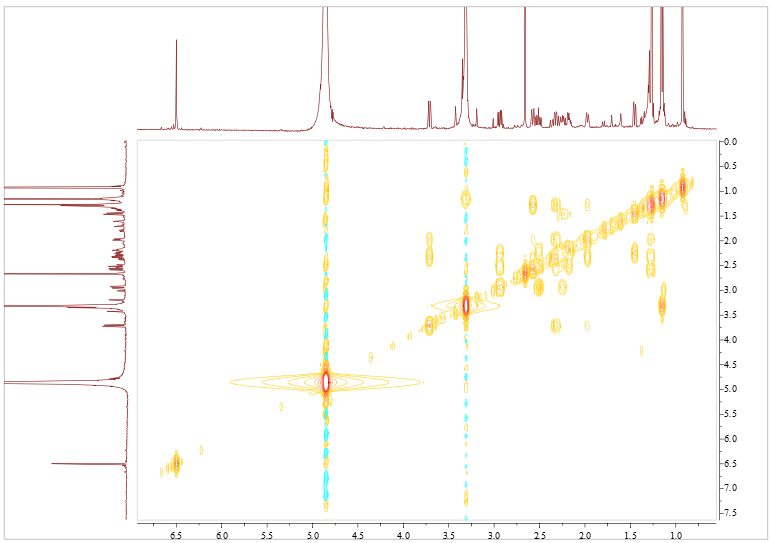


**Figure S17.** ^1^H-^1^H COSY spectrum of the compound **3.**


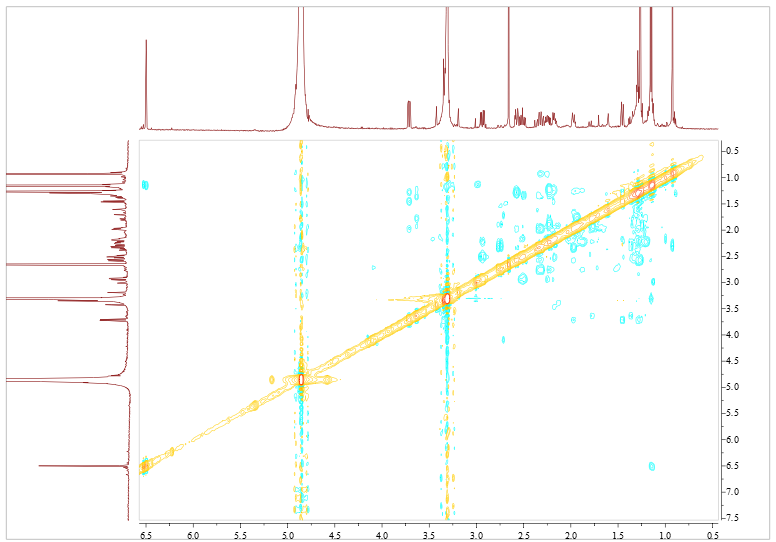


**Figure S18.**NOESY spectrum of the compound **3.**

**Figure S19.** HRESIMS spectrum of the compound **3.**
